# Cephalic Pancreaticoduodenectomy With Right Nephrectomy for Traumatic Ruptured Wilms Tumor

**DOI:** 10.7759/cureus.84959

**Published:** 2025-05-28

**Authors:** Elily D Apumayta, Nestor Sanchez, Eduardo Cayo, Marco Rioja, Enrique Franco

**Affiliations:** 1 Department of Abdominal Surgery, Instituto Nacional de Enfermedades Neoplásicas, Lima, PER; 2 Department of Abdominal Surgery/Abdominal Oncology Surgery, Instituto Nacional de Enfermedades Neoplásicas, Lima, PER; 3 Department of Radiology, Instituto Nacional de Enfermedades Neoplásicas, Lima, PER; 4 Department of Pathology, Instituto Nacional de Enfermedades Neoplásicas, Lima, PER; 5 Department of Urology/Urologic Oncology, Instituto Nacional de Enfermedades Neoplásicas, Lima, PER

**Keywords:** acute abdom, general trauma surgery, whipple’s pancreaticoduodenectomy, whipple surgery, wilms tumor

## Abstract

Wilms tumor is the most common pediatric extracranial solid tumor in Peru. Preoperative tumor rupture is an uncommon complication, and its management varies depending on the severity. If the bleeding is self-limited, does not affect hemodynamic stability, or is controlled with medical management, surgery may be delayed until after neoadjuvant chemotherapy.

This report describes a three-year-old male patient with a right Wilms tumor extending into the ipsilateral renal vein, accompanied by bulky retroperitoneal lymph nodes and multiple bilateral pulmonary metastases. He presented with severe abdominal pain and distension following abdominal trauma. Due to persistent hemodynamic decompensation after initial medical management, an emergency laparotomy was required. A devascularized duodenum adjacent to an area of extensive tumor bleeding confirmed a preoperative tumor rupture and a grade V duodenal injury. These findings necessitated a multivisceral emergency resection. The surgery included a right nephrectomy with en-bloc thrombectomy, resection of macroscopic lymph node metastases, and a cephalic pancreaticoduodenectomy.

Tumor rupture must be evaluated both clinically and radiologically to determine the safest course of treatment. Life-threatening hemodynamic decompensation unresponsive to medical management requires emergency surgery. Although multi-visceral resection is not the preferred approach for the initial management of Wilms tumor, it may become necessary due to injuries associated with tumor rupture. In such emergency oncologic surgeries, the selected techniques must uphold oncologic principles, minimize the risk of serious complications, and effectively address the underlying emergency.

## Introduction

Wilms tumor is the most common extracranial pediatric solid tumor in Peru [1]. It is typically diagnosed in children aged 2 to 5 years, with a slight female predominance. Approximately 50% of cases are non-metastatic at the time of diagnosis [2]. Treatment is guided by two main protocols: the American COG (Children's Oncology Group) and the European SIOP (International Society of Pediatric Oncology), which stratify patients into risk groups to assign a treatment plan based on the presence of prognostic factors.

In accordance with COG guidelines, upfront surgery is recommended for early-stage tumors suitable for R0 resection-macroscopic and microscopic complete excision. This approach should include thorough surgical staging and adequate lymph node sampling. It is also recommended to avoid resection of other organs and minimize the risk of tumor spillage. However, the strategy differs in cases of bilateral tumors, solitary kidneys, genetic predisposition syndromes, tumor thrombus in the inferior vena cava at or above the level of the hepatic veins, or symptomatic pulmonary metastases, which benefit from neoadjuvant chemotherapy given before surgery, the cornerstone of treatment [3]. Nonetheless, the surgical approach may vary in cases of tumor rupture.

Preoperative tumor rupture occurs in 2% to 5% of Wilms tumor cases, most commonly secondary to abdominal trauma or, less frequently, spontaneously. In the latter, it is more likely when the tumor volume exceeds 500 mL [2,4]. So, clinicians should suspect Wilms tumor rupture in cases presenting with sudden abdominal pain, distension, signs of shock, and a sharp hemoglobin drop, especially in the setting of trauma or large tumor burden. The extent of rupture and bleeding correlates with the clinical presentation and influences the treatment approach. A large tumor fissure with significant bleeding and hemodynamic decompensation unresponsive to medical management is the main indication for emergency surgery in this context [4]. Conversely, if the bleeding is limited and can be controlled medically, surgery may be postponed until after neoadjuvant chemotherapy.

On the other hand, cephalic pancreaticoduodenectomy in this age group is rare due to the low incidence of primary pancreatic neoplasms. An average incidence of 2.5 cases per hospital has been reported [5]. The primary diagnoses treated with this surgery include the solid pseudopapillary neoplasm of the pancreas (Frantz tumor), followed by pancreatoblastoma, carcinoma, and benign primary tumors. A multicenter series reports that more than half of the patients require postoperative intensive care unit (ICU) care, and approximately 1% undergo vascular reconstruction due to vessel involvement [5].

At our hospital, approximately 20 major urological surgeries for Wilms tumor are performed annually, with one typically related to tumor rupture. This report presents an emergency surgical case of Wilms tumor rupture complicated by duodenal necrosis after blunt abdominal trauma, managed with a rare pediatric multi-visceral resection, including pancreaticoduodenectomy.

## Case presentation

A three-year-old male patient with no significant medical history was diagnosed with clinical stage IV right Wilms tumor with multiple lung metastases. The diagnosis was made based on the typical clinical presentation without the need for a biopsy. The primary tumor had replaced the entire right kidney and contained areas of necrosis, accompanied by a renal vein thrombus and retroperitoneal lymphadenopathy measuring up to 3 cm. The lung metastases were diffuse and bilateral, with the largest lesion measuring 9 cm in the right lower lobe.

**Figure 1:**
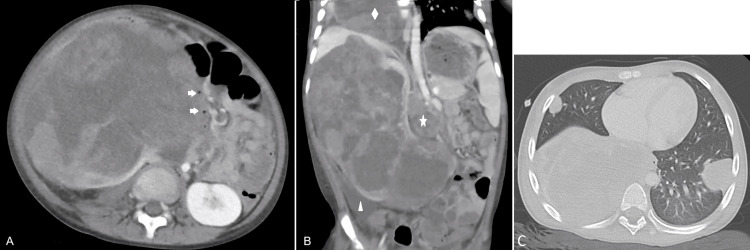
Preoperative CT scan CT: Computed tomography Figure 1. Right renal solid tumor with heterogeneous enhancement, including necrotic and hemorrhagic areas, and lung metastases A. Axial plane: The tumor compresses the inferior vena cava medially and displaces retroperitoneal structures. The duodenum is shifted to the left, nearly flattened, and contains blood with an air-fluid level (indicated by arrows). B. Coronal plane: Presence of precaval lymphadenopathy (indicated by a star). Free pelvic fluid with a density of 40 HU (triangle). The largest pulmonary metastasis measuring up to 9 cm in the right lower lobe (diamond). C. Thorax axial plane: Bilateral pulmonary metastases.

On the third day after receiving the first course of a high-risk chemotherapy regimen consisting of vincristine, actinomycin, and doxorubicin, the patient experienced blunt abdominal trauma from falling down the stairs, followed by severe abdominal pain and a drop in hemoglobin from 9 g/dL to 5.6 g/dL. A computed tomography (CT) scan revealed tumor rupture, evidenced by the presence of free peritoneal fluid with hematic density and intratumoral hemorrhagic areas. The duodenum was displaced and transversely distended with blood content (Figure 1). Despite resuscitation efforts, the patient remained hemodynamically unstable and underwent emergency surgery. 

**Figure 2:**
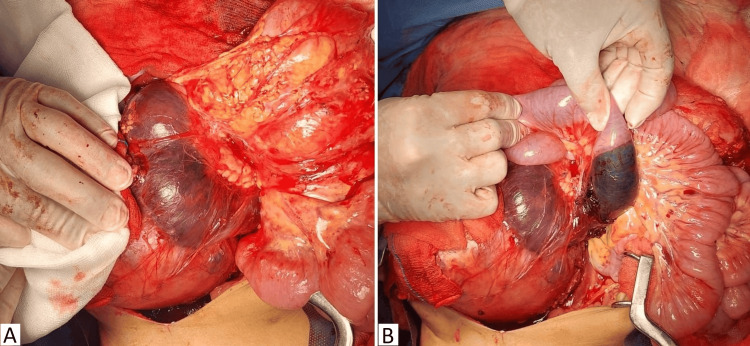
Intraoperative findings A. Extensive tumor replacing the right kidney. The tumor capsule is ruptured on its medial and anteromedial aspects and covered with gauze as a bleeding control measure. The duodenum is displaced, devascularized, strongly adhered to the tumor, inflated with blood, and tense surface. B. The second, third, and fourth portions of the duodenum appear devascularized, purplish, and filled with blood.

Through an extended supraumbilical midline incision, approximately 1000 cc of hemoperitoneum was identified. The right kidney tumor was ruptured along its anterior and anteromedial aspects. It was infiltrating the perinephric fat and was firmly adherent to the second and third portions of the duodenum, which appeared devitalized, violaceous, and contained blood. The fourth portion of the duodenum was also devitalized and contained blood (Figure 2). A Daum type I vascular thrombus was present. The pancreas was firm, with a 1 mm Wirsung duct.

**Figure 3:**
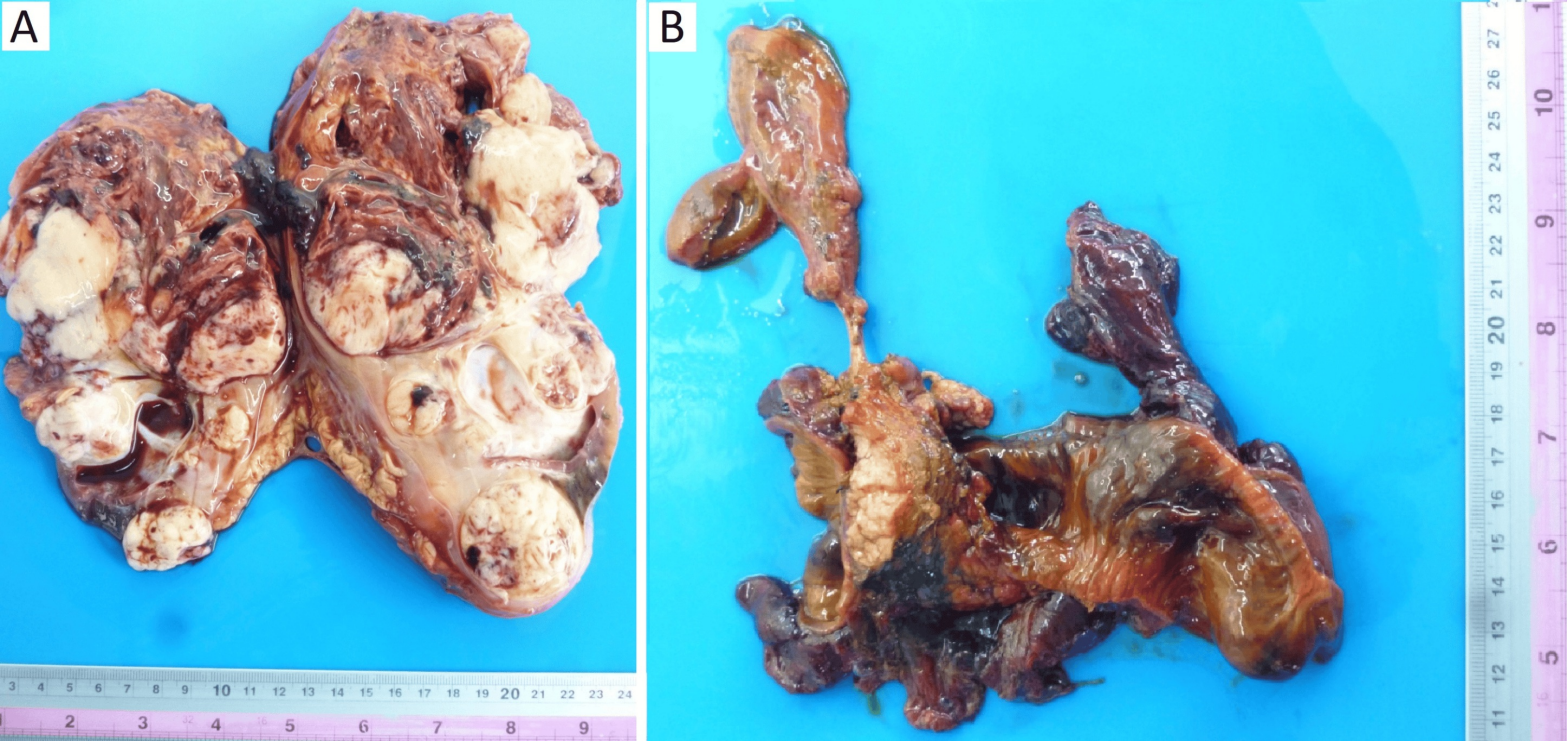
Macroscopic findings A. Right nephrectomy specimen: Right renal tumor measuring 20 × 11 × 10 cm, multinodular with hemorrhagic and necrotic areas, and a rupture site measuring 12 × 8 cm. B. Cephalic pancreaticoduodenectomy specimen: Duodenum with congested mucosa and wall bulging due to a hematoma involving the full thickness. The serosal surface appears necrotic and hemorrhagic. The pancreas and gallbladder show no significant alterations.

A right radical nephrectomy was performed along with an en-bloc renal vein thrombectomy and a cephalic pancreatoduodenectomy with pylorus preservation and level I mesopancreatic resection (Figure 3). 

**Figure 4:**
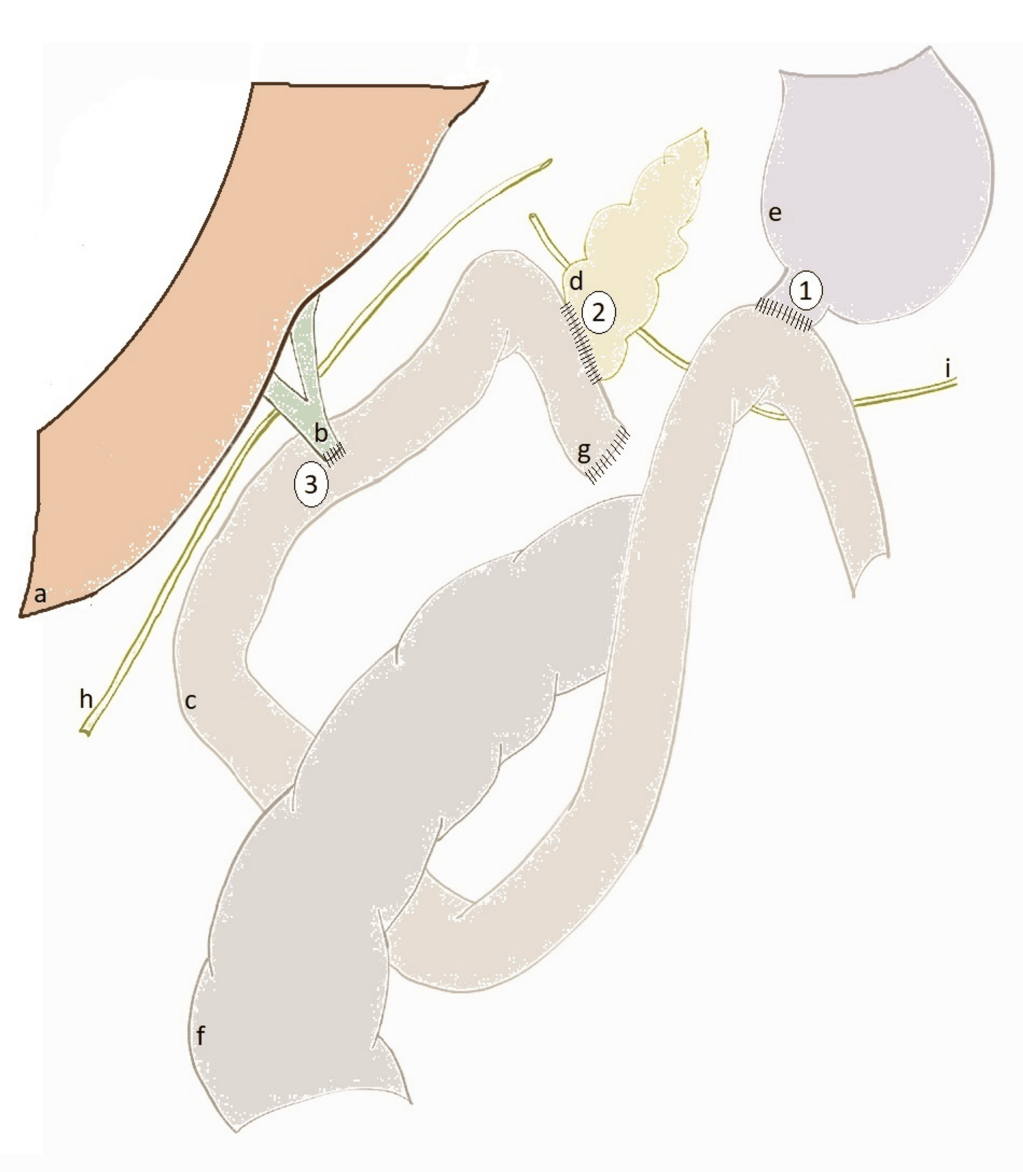
Reconstruction 1. End-to-side duodenojejunal anastomosis. 2. End-to-side invaginating pancreatojejunal anastomosis. 3. End-to-side hepaticojejunal anastomosis. a: Liver, b: common biliary duct, c: jejunum, d: pancreas stump, e: stomach, f: colon, g: jejunal stump, h: anterior laminar drain, i: posterior laminar drain. The image is created by the author.

Reconstruction was carried out using the Child technique, with transmesocolic jejunal ascent, an invaginating end-to-side pancreaticojejunal anastomosis, and an end-to-side hepatojejunal anastomosis supported by external stenting. Perianastomotic laminar drains were placed (Figure 4).

By postoperative day four, the patient had recovered from shock, and mechanical ventilation was discontinued. On postoperative day 10, chemotherapy was resumed with vincristine monotherapy at 1.5 mg/m² for two weekly cycles due to the progression of pulmonary metastases. The patient experienced delayed gastric emptying, which was managed successfully with medical treatment. The total ICU stay lasted 60 days. During this period, chemotherapy was continued with a regimen of irinotecan 20 mg/m² and vincristine 1.5 mg/m². The COG UH-1 chemotherapy protocol for his clinical stage has been reinitiated based on vincristine, doxorubicin, cyclophosphamide, carboplatin, and etoposide. Radiotherapy is included in the treatment plan.

**Figure 5:**
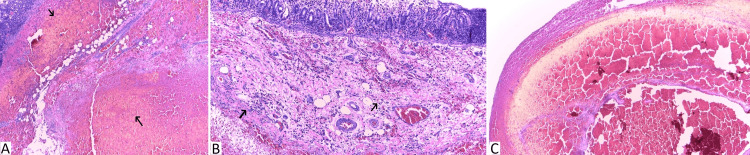
Microscopic findings A. H&E staining at 400x magnification. Organized hematoma (black arrows) involving the mucosa, submucosa, and muscularis propria. B. H&E staining at 400x magnification. Inflammatory infiltrate within the mucosa (black arrows). C. H&E staining at 100x magnification. Distortion of the normal architecture of the duodenal wall.

Pathological examination revealed a unifocal mixed nephroblastoma, composed of 40% blastemal, 40% epithelial, and 20% stromal components, with diffuse anaplasia. No nephrogenic rests were identified. The tumor showed invasion of the renal sinus and vein, with extension beyond the renal capsule. Six out of ten resected lymph nodes were involved by the neoplasm. The vascular margin was positive. The duodenal mucosa appeared congested and hemorrhagic, with an extensive hematoma involving the submucosa and muscularis and extending to the subserosa (Figure 5).

## Discussion

Preoperative rupture of Wilms tumor is uncommon, occurring in approximately 3% of cases [2,4]. Rupture may result in bleeding within the peritoneal, retroperitoneal, or intratumoral spaces and must be distinguished from tumor spillage. Tumor rupture with spillage into the peritoneal cavity is classified as clinical stage III, as it negatively impacts prognosis. Consequently, oncologic treatment must include triple-agent chemotherapy followed by a 15/10.8 Gy abdominal radiation therapy dose [6]. Tumor rupture typically presents with acute abdominal pain and/or a drop in hemoglobin. In cases of localized bleeding, accounting for 29% of tumor ruptures, symptoms may be limited to nausea, vomiting, and loss of appetite, and treatment may not be required [4,6].

The initial approach to tumor rupture is medical management. It starts with intravenous fluids, blood transfusions, and monitoring vital signs. If the patient is stabilized, chemotherapy may be continued and surgery postponed to minimize the risk of further tumor contamination within the abdominal cavity and for downsizing the tumor [4]. Delayed surgery following tumor rupture has been reported in up to 66% of cases, typically after 1 to 20 weeks of chemotherapy, even in extensive rupture without localization [6]. However, tumor rupture remains the leading cause of emergency surgery in patients diagnosed with Wilms tumor [7].

Our patient experienced blunt abdominal trauma from falling down the stairs, presenting with severe abdominal pain, distension, and signs of peritoneal irritation, along with a hemoglobin drop of 4 g/dL. This clinical picture was consistent with tumor rupture, resulting in hypovolemic shock unresponsive to medical management. The severity of the rupture in this case may be associated with the tumor’s size, with a maximum diameter exceeding 20 cm, which can predispose even to spontaneous rupture. Although tumors of similar dimensions do not always rupture, the average volume in reported cases of Wilms tumor rupture is approximately 533 cm³ [6].

Usually, the retroperitoneal location of the duodenum protects it from trauma. However, that severe force exerted to the right abdomen, over a previously displaced duodenum, also resulted in duodenal injury. During exploratory laparotomy, duodenal devascularization was identified. Microscopic examination revealed necrotic and erosive duodenal mucosa, with an organized hematoma in the deeper layers. The grade V duodenal injury, in association with tumor rupture, warranted resection of the duodenopancreatic complex. In children, blunt duodenal trauma is more common than open trauma and is typically associated with other injuries [8].

Imaging played a crucial role in establishing the diagnosis. Although ultrasound may reveal some indicative findings, computed tomography is the most valuable modality for detecting both Wilms tumor rupture and duodenal trauma [8,9]. The primary radiological sign of tumor rupture is ascites located posterior to the cul-de-sac. Additional findings may include poor tumor demarcation, subcapsular, perinephric, or retroperitoneal fluid collections, ipsilateral pleural effusion, or evidence of tumor fracture communicating with free peritoneal fluid. In cases of chronic bleeding, an encapsulated retroperitoneal effusion may be observed [6,9]. However, up to 21.4% of patients may lack definitive radiological signs [4]. In our patient, clear signs of tumor rupture were present (Figure 1). The duodenum preoperatively was not normal. It was displaced, exhibited a heterogeneous density, and was filled with fluid. There was not a gross thickening of the duodenal wall, nor extraluminal air, that could indicate duodenal hematoma or laceration, respectively. Even though a devascularization of the duodenum was not clearly stated due to its rarity, the preoperative plan included the exploration of the descending and transverse portions of the duodenum and pancreatic head, beyond the nephrectomy. 

According to the above, surgery was initiated through a midline incision with a right nephrectomy via a transperitoneal approach to control the bleeding. We opted for a midline incision instead of the traditional transverse abdominal incision due to its shorter operation time and optimal exposure with minimal trauma in correspondence to the multi-visceral trauma condition with hemodynamic instability. Kidney mobilization was challenging due to the involvement of perirenal fat, particularly on the posterior surface. Subsequently, a cephalic pancreaticoduodenectomy was performed using a posterior approach. The pylorus was preserved to reduce operative time, minimize intraoperative bleeding, and prevent biliary reflux. A level I mesopancreatic resection was carried out, as the underlying etiology differed from that of periampullary malignancies. Given the 1-millimeter Wirsung duct and the emergency context, an invagination technique was chosen over the classic duct-to-mucosa pancreaticojejunostomy due to its easier handling and shorter operative time.

Although the ISGPS (International Study Group of Pancreatic Surgery) recommends duct-to-mucosa pancreaticojejunostomy in the presence of high-risk features for postoperative pancreatic fistula-such as pancreatic ducts less than 3 mm in diameter [10]-some authors advocate for the invagination technique in cases involving a soft pancreas with a small, unobstructed duct [11]. Nevertheless, this technique may not be suitable for large pancreatic remnants or when the pancreatic stump does not align well with the jejunal lumen. Perianastomotic intraperitoneal drains were also placed. Perioperative medications such as octreotide or hydrocortisone, which are sometimes used to reduce the risk of postoperative pancreatic fistula, were not administered. In accordance with our institutional protocol, the presence of pancreatic fistula was ruled out by evaluating the relationship between serum and drain amylase levels, which was negative on postoperative day three.

The patient developed grade C delayed gastric emptying as a late postoperative complication after postoperative day 14. A mechanical cause was ruled out via upper endoscopy, which confirmed a patent pylorus. Consequently, the patient was treated with prokinetics and received parenteral nutrition for over three weeks. Relaparotomy was not required. Pylorus-preserving pancreaticoduodenectomy, as performed in our patient, has not been shown to reduce the incidence of this or other short- and long-term complications [5,12,13].

Recommendations for Whipple surgery in children are largely extrapolated from the adult population, where the incidence of pancreatic neoplasia is nine times higher. There are no established guidelines favoring alternative techniques over the classic Whipple procedure, nor specific recommendations regarding reconstruction or anastomosis methods in pediatric cases. Nonetheless, surgical experience remains a critical factor. Several series have demonstrated that this procedure is safe in children, with outcomes comparable to, or even better than, those observed in adults [5,14]. In our patient, the only postoperative complication related to the procedure was delayed gastric emptying, which was successfully managed with medical treatment.

Regarding oncological outcomes, tumor rupture with spillage has not been shown to significantly affect overall survival, but it does impact disease-free survival. Recurrence or metastasis occurs in 24% of tumor rupture cases, compared to 7.3% in patients without rupture [6]. Among patients with tumor rupture, the five-year local control rate varies depending on the extent of bleeding, 83% for rupture into the peritoneal cavity versus 100% for retroperitoneal involvement [15]. That is why radiotherapy has been planned for local control; however, it could not be administered as usual on postoperative days 10 to 14 due to the patient’s ICU stay. It is still scheduled to proceed once the patient has improved his respiratory function. Although he continued with systemic therapy, he had a progression of lung metastases. This will be a key determinant of prognosis. The patient remains under systemic treatment for four months. 

## Conclusions

We present the case of a three-year-old male patient who required emergency surgery, including a right nephrectomy and cephalic pancreaticoduodenectomy, due to abdominal trauma that resulted in tumor rupture and severe duodenal injury. This case highlights three important points. The importance of rapid multidisciplinary intervention in Wilms tumor rupture with adjacent organ injury supports the feasibility of emergency Whipple procedures in experienced pediatric surgical centers. Also, the oncologic significance of Wilms tumor rupture, associated with a higher risk of postoperative recurrence or metastases, supports the aggressive treatment.

Although multi-visceral resection is not ideal in upfront surgery for Wilms tumor, the life-threatening nature of active bleeding due to tumor rupture, along with duodenal devascularization, necessitated the surgical approach described. While emergency cephalic pancreaticoduodenectomy is rare in pediatric oncologic surgery, we recommend pyloric preservation and invagination pancreaticojejunostomy due to their shorter operative time and ease of handling, respectively. The placement of drains is also important for monitoring signs of pancreatic fistula and other postoperative complications. Despite implementing the best advice available, delayed gastric emptying was observed and resolved with medical management, without any long-term implication. Tumor rupture must be evaluated both clinically and radiologically to ensure the safest treatment approach. Life-threatening hemodynamic decompensation unresponsive to medical management requires emergency surgical intervention. In such cases of emergency oncologic surgery, the selected techniques should adhere to oncological principles, aim to minimize the risk of serious complications, and effectively address the underlying emergency.
